# Continuous Medicaid Eligibility During the COVID-19 Pandemic and Postpartum Coverage, Health Care, and Outcomes

**DOI:** 10.1001/jamahealthforum.2024.0004

**Published:** 2024-03-08

**Authors:** Jamie R. Daw, Colleen L. MacCallum-Bridges, Katy B. Kozhimannil, Lindsay K. Admon

**Affiliations:** 1 Department of Health Policy and Management, Columbia University Mailman School of Public Health, New York, New York; 2 Department of Obstetrics and Gynecology, University of Michigan, Ann Arbor; 3 Division of Health Policy and Management, University of Minnesota School of Public Health, Minneapolis

## Abstract

**Importance:**

Pursuant to the Families First Coronavirus Response Act (FFCRA), continuous Medicaid eligibility during the COVID-19 public health emergency (PHE) created a de facto national extension of pregnancy Medicaid eligibility beyond 60 days postpartum.

**Objective:**

To evaluate the association of continuous Medicaid eligibility with postpartum health insurance, health care use, breastfeeding, and depressive symptoms.

**Design, Setting, and Participants:**

This cohort study using a generalized difference-in-differences design included 21 states with continuous prepolicy (2017-2019) and postpolicy (2020-2021) participation in the Pregnancy Risk Assessment Monitoring System (PRAMS).

**Exposures:**

State-level change in Medicaid income eligibility after 60 days postpartum associated with the FFCRA measured as a percent of the federal poverty level (FPL; ie, the difference in 2020 income eligibility thresholds for pregnant people and low-income adults/parents).

**Main Outcomes and Measures:**

Health insurance, postpartum visit attendance, contraceptive use (any effective method; long-acting reversible contraceptives), any breastfeeding and depressive symptoms at the time of the PRAMS survey (mean [SD], 4 [1.3] months postpartum).

**Results:**

The sample included 47 716 PRAMS respondents (64.4% aged <30 years; 18.9% Hispanic, 26.2% non-Hispanic Black, 36.3% non-Hispanic White, and 18.6% other race or ethnicity) with a Medicaid-paid birth. Based on adjusted estimates, a 100% FPL increase in postpartum Medicaid eligibility was associated with a 5.1 percentage point (pp) increase in reported postpartum Medicaid enrollment, no change in commercial coverage, and a 6.6 pp decline in uninsurance. This represents a 40% reduction in postpartum uninsurance after a Medicaid-paid birth compared with the prepolicy baseline of 16.7%. In subgroup analyses by race and ethnicity, uninsurance reductions were observed only among White and Black non-Hispanic individuals; Hispanic individuals had no change. No policy-associated changes were observed in other outcomes.

**Conclusions and Relevance:**

In this cohort study, continuous Medicaid eligibility during the COVID-19 PHE was associated with significantly reduced postpartum uninsurance for people with Medicaid-paid births, but was not associated with postpartum visit attendance, contraception use, breastfeeding, or depressive symptoms at approximately 4 months postpartum. These findings, though limited to the context of the COVID-19 PHE, may offer preliminary insight regarding the potential impact of post-pandemic postpartum Medicaid eligibility extensions. Collection of longer-term and more comprehensive follow-up data on postpartum health care and health will be critical to evaluating the effect of ongoing postpartum policy interventions.

## Key Points

**Question:**

What is the association between continuous Medicaid eligibility under the Families First Coronavirus Response Act and postpartum health insurance, postpartum visit attendance, contraception use, depressive symptoms, and breastfeeding?

**Findings:**

In this cohort study including 47 716 participants, continuous Medicaid eligibility was associated with large increases in postpartum Medicaid and decreased uninsurance for people with Medicaid-paid births. No changes were observed in other outcomes.

**Meaning:**

Continuous Medicaid eligibility during the COVID-19 pandemic significantly reduced loss of Medicaid after birth, suggesting similar uninsurance reductions may be expected from postpandemic postpartum Medicaid extensions, which most states plan to implement.

## Teaser

This cohort study evaluates the association of continuous Medicaid eligibility with postpartum health insurance, health care use, breastfeeding, and depression.

## Introduction

Pregnancy-related morbidity and mortality rates are high and rising in the US, particularly during the postpartum year.^1^ Health insurance may play a critical role in facilitating access to timely care to manage physical and mental health conditions after childbirth.^2,3,4,5^ Federal law requires all states to provide pregnancy Medicaid to low-income people from conception to 2 calendar months, or roughly 60 days, after delivery.^6^ To maintain Medicaid coverage after this time, postpartum people must qualify based on state-specific income eligibility rules for low-income adults/parents, which are typically less generous than those for pregnant people. As a result, from 2016 to 2018, nearly a quarter of people with Medicaid-paid births became uninsured by 4 months postpartum, representing approximately 330 000 live births annually.^7^

To improve postpartum coverage for Medicaid beneficiaries, the 2021 American Rescue Plan Act (ARPA) offered a new state option to use federal matching funds to extend pregnancy-related Medicaid eligibility to 1 year postpartum. As of January 2024, 43 states and Washington, DC, had adopted this option.^8^ COVID-19 pandemic response legislation could provide early insight into the potential effect of postpandemic ARPA extensions. The 2020 Families First Coronavirus Response Act (FFCRA) included a maintenance of effort (MOE) requirement preventing states from disenrolling Medicaid recipients during the federal public health emergency (PHE) from January 31, 2020, to May 11, 2023.^9^ This created a de facto national extension of pregnancy-related Medicaid eligibility. The objective of this cohort study was to estimate the association of continuous Medicaid eligibility under the FFCRA with postpartum health insurance, postpartum visit attendance, contraception, breastfeeding, and depressive symptoms.

## Methods

### Data Source

We used 2017 to 2021 data from the Centers for Disease Control and Prevention (CDC) Pregnancy Risk Assessment Monitoring System (PRAMS). PRAMS is a representative survey of a stratified random sample of postpartum people drawn monthly from state birth certificates. A detailed overview of the PRAMS methodology is available elsewhere.^10^ The University of Michigan institutional review board deemed this study of deidentified survey data exempt from review. We followed the Strengthening the Reporting of Observational Studies in Epidemiology (STROBE) reporting guidelines.

### Sample

We included PRAMS respondents for whom Medicaid was the primary payer on the birth certificate. To ensure that differences in state inclusion in PRAMS did not bias estimates of outcome changes over time, we limited the sample to the 25 jurisdictions with complete PRAMS data from 2017 to 2021. To avoid confounding of the FFCRA with other Medicaid policy changes, we further excluded 4 states that either expanded Medicaid under the ACA (Virginia, Utah, Michigan) or significantly increased pregnancy Medicaid eligibility (Missouri) during the study period. This resulted in the inclusion of 21 jurisdictions (20 states and New York City) (eTable 1 in Supplement 1).

### Outcome Measures

We evaluated 6 self-reported primary postpartum outcomes measured at the time of the PRAMS survey (mean [SD], 4 [1.3] months postpartum). For nearly all respondents, the survey was completed after the end of pregnancy Medicaid eligibility at 2 months/60 days postpartum (97% completed ≥3 months postpartum; 62% ≥ 4 months; 49% ≥ 5 months). Postpartum health insurance was hierarchically assigned as Medicaid, commercial (including military coverage), or uninsured. We also measured routine postpartum visit attendance, effective contraception use (vasectomy, tubal ligation, contraceptive injection, pill, patch, ring, intrauterine device [IUD] or implant), long-acting reversible contraceptive (LARC) use (IUDs or implants), current breastfeeding, and depressive symptoms (score ≥3 on the PRAMS modified Patient Health Questionnaire-2 [PHQ-2], indicating a likely diagnosis of major depression^11^). We hypothesized that continuous Medicaid eligibility would increase Medicaid enrollment, thereby improving access to services such as family planning, treatment for breast infections, lactation consulting, and mental health care, which could in turn increase breastfeeding rates and reduce depressive symptoms.^12^ Because routine postpartum visits typically occur 6 to 8 weeks after birth—before pregnancy Medicaid eligibility ends—we did not hypothesize a change; however, attendance could increase because of the wider time window in which patients had coverage and could schedule this visit.

### Study Design and Statistical Analysis

**Figure 1.**
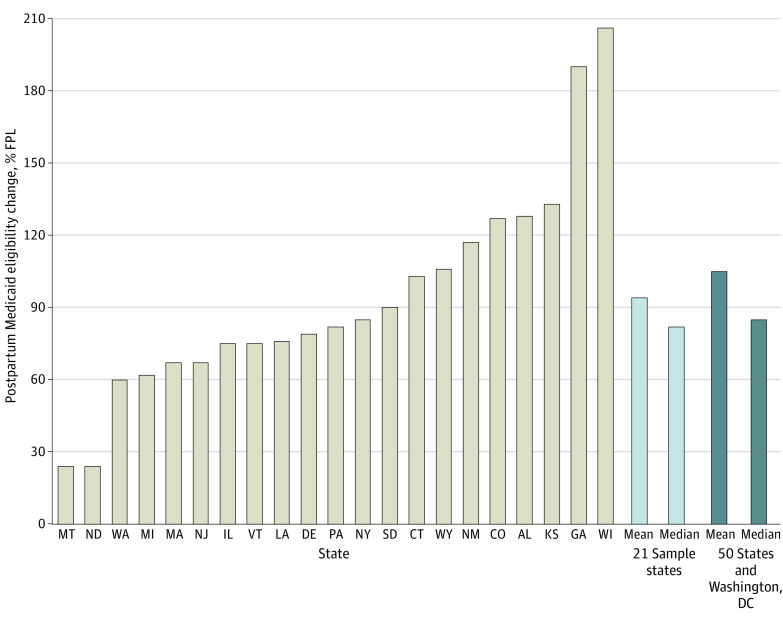
Variation in the Families First Coronavirus Response Act (FFCRA)*-*Associated Postpartum Medicaid Eligibility Change Among 21 Sample States The FFCRA-associated postpartum Medicaid eligibility change is the difference in the income eligibility level for pregnancy Medicaid and for low-income adults/parents in each state in 2020. This change represents the effective increase in the Medicaid income eligibility level after 60 days postpartum that resulted from the maintenance of effort provision of the FFCRA. States with a larger eligibility increase had more people who became newly eligible for continued Medicaid after pregnancy (and thus did not lose Medicaid eligibility postpartum) during the COVID-19 public health emergency. eTable 1 in Supplement 1 provides additional details on state-level income eligibility criteria. FPL indicates federal poverty level.

As of January 31, 2020, the FFCRA effectively raised each state’s income eligibility criteria for postpartum parents/adults (mean in the sample states, 114% of the federal poverty level [FPL]) to that of pregnant people (mean in the sample states, 208% FPL). Although the FFCRA is a federal policy with no state variation, the state policy intensity of the FFCRA varied widely based on differences in states’ prepolicy Medicaid eligibility criteria for pregnancy and low-income parents/adults. Among the 21 included jurisdictions, the FFCRA-associated postpartum Medicaid eligibility increase ranged from 24% FPL in Montana to 206% FPL in Wisconsin (mean, 94%, median: 82%), which was similar to the distribution for all 50 states and Washington, DC (range, 0%-284%; mean, 105%; median, 85%) (Figure 1; eTable 1 in Supplement 1). States with a larger eligibility increase had more people who became newly eligible for continued Medicaid after pregnancy (and thus did not lose Medicaid eligibility at 60 days postpartum) during the PHE. We used a generalized difference-in-differences analysis that exploited this state-level variation to estimate the association between the FFCRA-associated gain in postpartum Medicaid eligibility and changes in the outcomes of interest.

We estimated survey-weighted linear regression models for each outcome where the primary independent variable was an interaction between a post-FFCRA policy indicator (2020-2021) and the state-level FFCRA-associated change in postpartum Medicaid eligibility measured as a percent of the FPL. For interpretability, we scaled regression estimates to represent the prepolicy to postpolicy percentage point (pp) change in each outcome associated with a 100% FPL increase in the postpartum Medicaid eligibility threshold (similar to the national mean change associated with the FFCRA). To provide an estimate of the relative effect of the policy, we calculated percent changes from baseline by dividing the scaled regression estimates by the prepolicy (2017-2019) mean for each outcome.

All models included state and year fixed effects to control for time-invariant outcome differences across states and common time effects across all states. In adjusted models, we included individual-level covariates measured in PRAMS (race and ethnicity, age, education, rural/urban residence, marital status, survey language, parity, chronic conditions, household income as a percent of FPL, and postpartum month of survey completion). To capture any differential associations of COVID-19 across states and time, we adjusted for state-month level unemployment rate; COVID-19 case and death rate; and indicators for whether a stay-at-home order, Marketplace special enrollment period, or elective medical procedures suspension order was in place (eTable 7 in Supplement 1). We calculated heteroskedasticity-robust standard errors clustered at the state level to account for within-state error correlation and the state-level exposure variable.^13^ We applied CDC PRAMS weights to account for the sampling design and nonresponse.^10^

We also conducted 3 secondary analyses. First, we estimated stratified models for the 3 largest race and ethnicity groups represented in PRAMS (Hispanic, non-Hispanic Black [Black hereafter], and non-Hispanic White [White hereafter] participants; measured through the birth certificate). Population-level interventions intersect with existing institutions and structures, and thus, even universal policies may influence racial disparities.^14,15,16,17^ Indeed, prior studies of Medicaid expansions and perinatal coverage have found differential effects by race and ethnicity.^18^ Given that Black and Hispanic birthing people are disproportionately covered by Medicaid,^19,20^ and disproportionately live in states with less generous low-income parent/adult Medicaid eligibility,^21^ we hypothesized that the FFCRA could disproportionally affect Black and Hispanic individuals. However, these stratified results should be considered exploratory given the small sample sizes.

Second, we estimated models stratified by pre-FFCRA pregnancy Medicaid income eligibility criteria (<213% of the FPL vs ≥213% of the FPL, the median pregnancy Medicaid eligibility threshold among sample states). Third, we disaggregated postpartum contraception use into 5 methods: (1) tubal ligation, (2) short-acting hormonal, (3) LARC, (4) condoms only, and (5) no method or only a nonhormonal/nonbarrier method.

Causal interpretations of the study estimates require parallel trend assumptions, ie, that the outcome changes from prepolicy to postpolicy would have been the same across states if not for state-level differences in the FFCRA-associated change in postpartum Medicaid eligibility. To test this assumption, we conducted event studies estimating separate coefficients for the association between the FFCRA-associated eligibility change and each prepolicy and postpolicy year relative to 2019, the last prepolicy year (see eMethods in Supplement 1). We conducted analyses from June 2023 to December 2023 using Stata MP statistical software (version 17; Stata Corp) with statistical tests at the .05 significance level. Observations with missing outcome data were excluded from the respective analyses (mean, 2.2% missing across outcomes; range, 0.7%-4.1%). Observations with missing covariate data were included using a categorical missing indicator.

## Results

Of the 47 716 respondents with a Medicaid-paid birth, most were younger than 30 years (64.4%), 22.5% reported at least 1 chronic condition, and 36.3% identified as non-Hispanic White, 26.2% as Black, and 18.9% as Hispanic (Table 1). Sample characteristics were largely consistent from the prepolicy to the postpolicy periods.

**Table 1.** Sample Characteristics, Postpartum PRAMS Respondents With Medicaid-Paid Births^a^

Characteristic	No. (%)		
	Overall, N = 47 716	Prepolicy (2017-2019), n = 29 900	Postpolicy (2020-2021), n = 17 816
Age, y			
<20	3519 (7.4)	2256 (7.5)	1263 (7.3)
20-24	12 009 (26.4)	7772 (27.2)	4237 (25.2)
25-29	14 474 (30.6)	9203 (30.7)	5271 (30.6)
30-34	10 923 (21.9)	6639 (21.8)	4284 (22.4)
>35	6791 (13.6)	4030 (12.9)	2761 (14.6)
Race and ethnicity^b^			
Asian	1930 (4.4)	1198 (4.5)	732 (4.1)
Black	13 514 (26.2)	8879 (26.4)	4635 (26.0)
Hispanic	8919 (18.9)	5475 (18.4)	3445 (19.7)
Multiple races	2410 (3.5)	1473 (3.5)	937 (3.5)
Native American	4095 (1.6)	2354 (1.5)	1741 (1.6)
Pacific Islander	146 (0.3)	83 (0.3)	63 (0.3)
White	12 136 (36.3)	7701 (36.9)	4435 (35.3)
Missing	1616 (1.2)	1000 (1.2)	616 (1.1)
Other non-White	2950 (7.7)	1738 (7.2)	1212 (8.4)
Education			
<High school	9915 (20.5)	6349 (21.0)	3566 (19.6)
High school	17 753 (39.9)	11 021 (39.2)	6732 (41.0)
>High school	19 605 (38.7)	12 275 (38.8)	7330 (38.5)
Household income (% FPL)			
<100	20 321 (39.2)	13 562 (41.7)	6759 (35.0)
100-149	8465 (18.1)	5229 (17.8)	3236 (18.6)
150-204	5314 (11.6)	3388 (12.0)	1926 (10.9)
>205	5958 (13.2)	3218 (11.4)	2740 (16.3)
Missing	7658 (17.9)	4503 (17.0)	3155 (19.3)
Married	16 136 (35.5)	10 159 (35.8)	5977 (34.9)
Rural resident	10 378 (15.2)	6376 (15.4)	4002 (14.9)
Survey language			
English	42 341 (86.9)	26 615 (87.2)	15 726 (86.4)
Spanish	5192 (12.4)	3158 (12.0)	2034 (13.1)
Chinese	183 (0.7)	127 (0.7)	56 (0.5)
Primiparous	15 571 (33.2)	9775 (33.2)	5796 (33.1)
Any prepregnancy chronic conditions^c^	12 109 (22.5)	7171 (21.7)	4938 (23.9)
Hypertension	3531 (6.4)	2168 (6.3)	1363 (6.5)
Diabetes	1868 (3.4)	1152 (3.4)	716 (3.3)
Depression	9261 (17.6)	5418 (16.8)	3843 (18.8)

Abbreviations: FPL, federal poverty level; PRAMS, Pregnancy Risk Assessment Monitoring System.

^a^ Estimates shown are unweighted frequencies and survey-weighted percentages. Missing not shown for variables with less than 1% missing values.

^b^ Asian includes Chinese, Japanese, and other Asian. Pacific Islander includes Hawaiian and Filipino. All other categories are stated as they were listed in the PRAMS survey. In our subsequent regression analyses, we collapse race and ethnicity into 5 levels (Black non-Hispanic, white non-Hispanic, Hispanic, other, missing).

^c^ Self-reported hypertension, diabetes, or depression in the 3 months prior to pregnancy.

Table 2 shows the aggregate rates, and changes, for each outcome in the prepolicy (2017-2019) and postpolicy periods (2020-2021). Among people with Medicaid-paid births, continued enrollment in Medicaid postpartum rose from 63.1% to 72.8% (9.7 pp increase; *P* < .001) and postpartum uninsurance declined from 16.7% to 9.6% (7.1 pp decrease; *P* < .001). Postpartum visit attendance (1.1 pp decrease), effective contraception (3.2 pp decrease), LARC use (1.9 pp decrease), and depressive symptoms (1.2 pp decrease) all decreased in the postpolicy period. Breastfeeding did not change. eFigure 1 in Supplement 1 shows scatterplots of the unadjusted state-level prepost policy changes in the outcomes and the FFCRA postpartum Medicaid eligibility change. Visually, larger state eligibility changes were correlated with larger increases in postpartum Medicaid enrollment and decreases in uninsurance; correlations for other outcomes appear null or small.

**Table 2.** Prepolicy to Postpolicy Changes in Postpartum Outcomes and Difference-in-Difference Estimates

Postpartum outcome	Prepolicy, % (2017-2019)	Postpolicy, % (2020-2021)	Percentage point change from prepolicy to postpolicy (95% CI)	Unadjusted difference-in-difference estimates, percentage point change associated with a 100% FPL increase in postpartum Medicaid eligibility (95% CI)	
				Unadjusted	Adjusted
Insurance status					
Medicaid	63.1	72.8	9.7 (8.4 to 11.0)	5.3 (2.7 to 7.9)	5.1 (1.8 to 8.4)
Commercial	20.2	17.6	−2.6 (−3.8 to −1.5)	1.2 (−3.6 to 6.0)	1.5 (−2.5 to 5.6)^a^
Uninsured	16.7	9.6	−7.1 (−9.0 to −6.1)	−6.5 (−13.1 to 0.1)	−6.6 (−13.5 to 0.3)
Postpartum visit	85.8	84.7	−1.1 (−2.1 to −0.06)	1.3 (−0.9 to 3.5)	0.8 (−1.4 to 3.0)
Effective contraception	56.8	53.6	−3.2 (−4.6 to −1.8)	−1.7 (−5.7 to 2.3)	−3.1 (−7.4 to 1.3)^a^
LARC	19.5	17.6	−1.9 (−3.0 to −0.8)	−0.4 (−2.2 to 1.3)	−0.9 (−2.8 to 1.0)
Still breastfeeding	38.1	39.3	1.2 (−0.2 to 2.5)	2.2 (−0.4 to 4.7)	2.8 (−0.2 to 5.8)^a^
Depressive symptoms	17.1	15.9	−1.2 (−2.2 to −0.2)	−2.1 (−6.8 to 2.5)	−1.9 (−5.9 to 2.1)

Abbreviations: FPL, federal property level; LARC, long-acting reversible contraception.

^a^ Event study analyses suggest the parallel-trend assumption may be violated. These results should be interpreted with caution.

**Figure 2.**
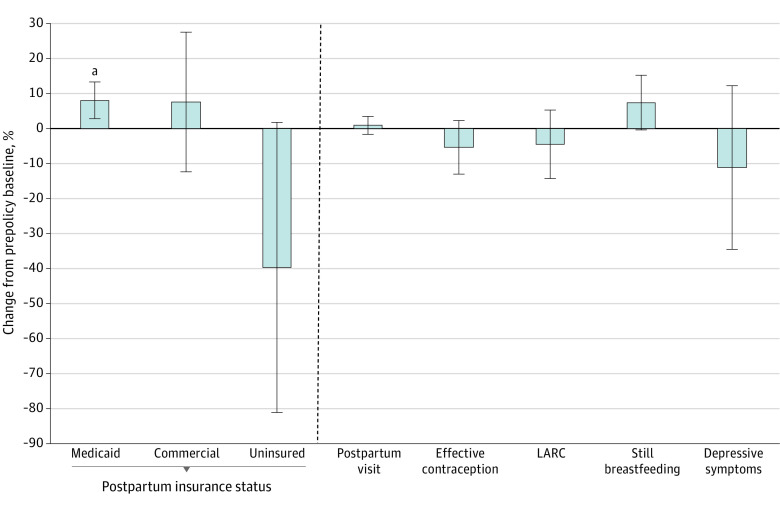
Percent Change in the Outcomes Associated With a 100% Federal Poverty Level (FPL) Increase in Postpartum Medicaid Eligibility Percent change is calculated as the adjusted difference-in-difference estimate for a 100% FPL change in postpartum Medicaid eligibility relative to the prepolicy baseline (2017-2019). Postpartum outcomes are measured at the time of the Pregnancy Risk Assessment Monitoring System survey (mean, 4 months after a live birth). Depressive symptoms are defined as a positive screen (score ≥3) on the PRAMS-modified Patient Health Questionnaire-2. LARC indicates long-acting reversible contraception including intrauterine devices and birth control implants. ^a^*P* = .004.

In adjusted difference-in-difference models, a 100% FPL increase in postpartum Medicaid eligibility was associated with a 5.1 pp increase in postpartum Medicaid coverage (95% CI, 1.8-8.4; 8.1% increase from prepolicy baseline), no change in commercial coverage (1.5 pp; 95% CI, −2.5 to 5.6), and a large but statistically nonsignificant −6.6 pp decrease (95% CI, −13.5 to 0.3; 40% decrease from prepolicy baseline) in uninsurance. Said another way, a 100% FPL increase in eligibility was associated with a significant reduction in postpartum Medicaid loss (−5.1 pp; 95% CI, −1.8 to −8.4) (Figure 2). Associations with other outcomes were small and not significant.

In exploratory analyses, we found large decreases in uninsurance among Black and White people with Medicaid-paid births (96.9% and 69.3% decline from baseline, respectively); but no measurable change for Hispanic respondents (eFigure 3 and eTable 4 in Supplement 1). We found larger increases in postpartum Medicaid in states with less generous (<213% FPL) compared to more generous (≥213% FPL) pre-FFCRA pregnancy eligibility (9.3 pp and 5.0 pp increases, respectively) (eTable 5 in Supplement 1). We did not find significant changes in use of specific contraceptive methods (eTable 6 in Supplement 1).

## Discussion

Using multistate representative survey data, we found that a 100% FPL increase in postpartum Medicaid income eligibility under the FFCRA was associated with an 8% increase in postpartum Medicaid coverage and a 40% reduction in uninsurance among those with a Medicaid-paid birth. These findings are consistent with studies using PRAMS and a similar approach to evaluate earlier expansions of Medicaid for low-income adults/parents.^22,23^ For example, Geiger et al^22^ and Wherry^23^ found that a 100% FPL increase in low-income adult/parent eligibility was associated with a 15 pp and 10 pp increase in preconception coverage, respectively. Our results are also consistent with health insurance increases documented during the COVID-19 PHE among female adults with children younger than year using the Current Population Survey^24^ and American Community Survey.^25^ Together, these findings suggest that policies extending Medicaid eligibility through the first year postpartum are likely to achieve the first-order goal of improving postpartum coverage and that states adopting the ARPA extension will continue to see significantly reduced levels of postpartum uninsurance. States with larger differentials in Medicaid eligibility for pregnant compared with nonpregnant adults, of which ACA nonexpansion states comprise a larger share, are likely to see the largest reductions in uninsurance, potentially reducing inequities across states in postpartum coverage.

Notably, even in the postpolicy period, we found that a substantial percentage (27%) of individuals who had Medicaid at delivery no longer reported Medicaid at the time of the PRAMS survey. Some Medicaid disenrollment reflects coverage switching—a similar proportion of people with Medicaid-paid births reported having commercial coverage postpartum before (20.2%) and after (17.6%) the FFCRA was in effect. Those reporting no coverage reduced significantly from 16.7% to 9.6% during the FFCRA; however, this still amounts to 1 in 10 individuals with a Medicaid-paid birth reporting postpartum uninsurance. This coverage loss could reflect that some individuals had only emergency Medicaid coverage for their birth and were not eligible for continuous coverage. We could not directly investigate this hypothesis because PRAMS does not include information on immigration status or emergency Medicaid. However, it is notable that we did not find any measurable improvements in uninsurance among Hispanic respondents associated with the FFCRA. Hispanic ethnicity is an imperfect proxy for immigration status. However, most people (80%) enrolled in emergency Medicaid are Latina^26^ and prior analyses of PRAMS have found that more than half (54.9%) of Spanish-speaking Hispanic individuals are uninsured postpartum after a Medicaid-paid birth, compared with 15.2% of non-Hispanic White individuals.^7^ Federal matching funds for state postpartum extensions exclude undocumented and recently documented immigrants. Given that 25% of US births are to an immigrant birthing parent,^27^ state decisions to use their own funds to cover immigrants will be an important determinant of the extent to which postpartum uninsurance is reduced nationally.

Reported postpartum uninsurance during the PHE could also reflect lack of beneficiary awareness of continued Medicaid coverage. Medicaid enrollment was underreported in national surveys relative to administrative data during the pandemic.^28^ There have also been documented instances of states sending erroneous notifications about coverage termination to postpartum Medicaid enrollees despite the FFCRA.^29^ States that are currently implementing postpandemic postpartum Medicaid extensions should invest in insurance navigators, enrollee outreach, and communication strategies to ensure beneficiary awareness of continued coverage and the benefits available in the postpartum year. Efforts may also be needed to ensure enrollees maintain Medicaid coverage if eligible, or switch to an affordable commercial plan, when pregnancy Medicaid coverage is terminated at 1 year after birth.

Despite documented gains in coverage, we did not identify significant associations between the FFCRA and postpartum visit attendance, contraception, breastfeeding, or depressive symptoms. Small or null effects on overall contraception and depressive symptoms were consistent with prior studies of Medicaid expansions.^30,31^ However, other studies have found increases in postpartum visit attendance and LARC use, which we did not identify.^30,31^ These null results could again reflect a lack of beneficiary awareness of continued coverage. It is also possible that Medicaid coverage may not have been enough to counteract the effects of the COVID-19 pandemic. Perinatal health care use decreased during the pandemic and a study of postpartum Medicaid beneficiaries in 2021 to 2022 found that being in quarantine or fear of COVID-19 infection were the most common reasons for not having a postpartum visit.^32,33^ We may also be underestimating the effect of the policy on postpartum outcomes due to the relatively short follow-up period for PRAMS respondents (mean: 4 months postpartum). The routine postpartum visit is typically scheduled for 6 to 8 weeks postpartum, and thus, may not be significantly affected by eligibility increases later in the postpartum period. Likewise, contraception is a major focus of the postpartum visit,^4^ which may limit the effecty of expanded coverage if initiation typically occurs prior to the loss of pregnancy Medicaid. Further, an additional 1 to 2 months of postpartum coverage may not be sufficient to affect breastfeeding continuation or mental health. This study cannot rule out positive effects on health care access and health outcomes in the later postpartum year. Yet, these null findings hold some lessons for ongoing implementation of postpartum Medicaid extensions in the postpandemic period. First, they highlight that translation of coverage to improved postpartum health care and health outcomes should not be assumed, and will likely require concerted efforts to improve health care access and delivery in the postpartum period, which is broadly recognized as deficient.^4,34,35^ For example, nearly 60% of people with a Medicaid paid birth report not having a usual source of care at 1 year postpartum.^33^ Unlike the FFCRA, state postpartum extensions will be implemented in a different clinical and policy context, with a clear focus on postpartum people, and states will have the opportunity to couple the extensions with other initiatives to improve postpartum care such as new payment models, programs to address social determinants of health, and enhanced coverage and/or reimbursement for nurse midwives and/or doulas.^36,37,38^

Finally, this analysis highlights how research on postpartum policy interventions is restricted by the limited data on postpartum outcomes, and the short-term follow-up period for these outcomes, in PRAMS and other available data sources.^39^ There is an urgent need for definitions of target postpartum outcomes and measurement of those outcomes in representative samples.^31,40^ Without better data, evaluating policies and programs aimed to improve postpartum health will be severely hindered, disrupting evidence-based policymaking, limiting opportunities to improve on policy implementation and potentially leading to false conclusions that interventions are not effective.

### Limitations

Apart from lacking data on outcomes beyond the early postpartum period, there are other limitations to this study. First, although the FFCRA-associated eligibility change in the 21 sample jurisdictions was similar to the national average, the results may not generalize to all states outside the sample. Second, the study design assumed that the outcomes would have continued along a similar trend (if not for the FFCRA) across states with differing FFCRA-associated eligibility changes. We found some evidence of differential prepolicy trends in commercial coverage, effective contraception, and breastfeeding, suggesting these results should be interpreted with caution (eFigure 2 and eTable 3 in Supplement 1). Finally, individuals suffering from depressive symptoms may have higher nonresponse to PRAMS. If this nonresponse varies by state eligibility change, it could potentially bias the results for depressive symptoms.

## Conclusions

This study found that continuous Medicaid eligibility during the COVID-19 PHE significantly reduced postpartum Medicaid loss but was not associated with postpartum visit attendance, contraception use, breastfeeding, or depressive symptoms in the early postpartum period. These findings, though potentially shaped by the COVID-19 pandemic and the fact that this policy was not tailored to postpartum people, may offer preliminary insights into the potential effects of postpandemic postpartum Medicaid eligibility extensions. More robust outcome data on health care use and well-being with longer-term follow-up in the postpartum year is urgently needed to inform and evaluate ongoing state adoption and implementation of postpartum Medicaid extensions.

## Notes

Supplement 1. eMethods eTable 1. FFCRA-associated postpartum Medicaid income eligibility changes by state eTable 2. Sample characteristics by tertile of FFCRA-associated postpartum Medicaid eligibility change for postpartum people with Medicaid-paid births eFigure 1. Unadjusted scatterplots and linear relationship between FFCRA postpartum Medicaid eligibility change and pre-post FFCRA postpartum outcomes by state among postpartum people with Medicaid-paid births eFigure 2. Event study plots eTable 3. F-tests for pre-period event study coefficients eTable 4. Difference-in-differences estimates stratified by race-ethnicity eFigure 3. Percent change in postpartum insurance status associated with a 100% FPL increase in postpartum Medicaid eligibility by race-ethnicity eTable 5. Difference-in-differences estimates stratified by pre-FFCRA income eligibility generosity for pregnant individuals eTable 6. Difference-in-differences estimates for additional contraceptive categories eTable 7. Definitions and data sources for state-month level COVID covariates eReferences

Supplement 2. Data Sharing Statement
