# Supplementary material for: Continuous Medicaid Eligibility During the COVID-19 Pandemic and Postpartum Coverage, Health Care, and Outcomes
Source: JAMA Health Forum. 2024 Mar 8;5(3):e240004. doi: 10.1001/jamahealthforum.2024.0004 (PMC10924249; doi:10.1001/jamahealthforum.2024.0004)
Supplement: Supplement 1. — eMethods eTable 1. FFCRA-associated postpartum Medicaid income eligibility changes by state eTable 2. Sample characteristics by tertile of FFCRA-associated postpartum Medicaid eligibility change for postpartum people with Medicaid-paid births eFigure 1. Unadjusted scatterplots and linear relationship between FFCRA postpartum Medicaid eligibility change and pre-post FFCRA postpartum outcomes by state among postpartum people with Medicaid-paid births eFigure 2. Event study plots eTable 3. F-tests for pre-period event study coefficients eTable 4. Difference-in-differences estimates stratified by race-ethnicity eFigure 3. Percent change in postpartum insurance status associated with a 100% FPL increase in postpartum Medicaid eligibility by race-ethnicity eTable 5. Difference-in-differences estimates stratified by pre-FFCRA income eligibility generosity for pregnant individuals eTable 6. Difference-in-differences estimates for additional contraceptive categories eTable 7. Definitions and data sources for state-month level COVID covariates eReferences [file jamahealthforum-e240004-s001.pdf]

Supplemental Online Content

Daw JR, MacCallum-Bridges CL, Kozhimannil KB, et al. Continuous Medicaid eligibility during the COVID-19 pandemic and postpartum coverage, health care, and outcomes. *JAMA Health Forum*. 2024;5(3):e240004. doi:10.1001/jamahealthforum.2024.0004

eMethods
eTable 1. FFCRA-associated postpartum Medicaid income eligibility changes by state
eTable 2. Sample characteristics by tertile of FFCRA-associated postpartum Medicaid eligibility change for postpartum people with Medicaid-paid births
eFigure 1. Unadjusted scatterplots and linear relationship between FFCRA postpartum Medicaid eligibility change and pre-post FFCRA postpartum outcomes by state among postpartum people with Medicaid-paid births
eFigure 2. Event study plots
eTable 3. F-tests for pre-period event study coefficients
eTable 4. Difference-in-differences estimates stratified by race-ethnicity
eFigure 3. Percent change in postpartum insurance status associated with a 100% FPL increase in postpartum Medicaid eligibility by race-ethnicity
eTable 5. Difference-in-differences estimates stratified by pre-FFCRA income eligibility generosity for pregnant individuals
eTable 6. Difference-in-differences estimates for additional contraceptive categories
eTable 7. Definitions and data sources for state-month level COVID covariates
eReferences

This supplemental material has been provided by the authors to give readers additional information about their work.

eMethods

a) Main Regression Specifications

To exploit the state-level variation in FFCRA policy intensity, the primary independent variable is the interaction between an indicator for the post-FFCRA policy period (2020-2021) and the FFCRA-associated change in the state Medicaid eligibility threshold for postpartum individuals, measured as the difference in the percent of the FPL eligibility threshold for pregnant women (the new *de facto* income limit for postpartum Medicaid beyond 60 days under the FFCRA) and low-income parents (the prior income limit for postpartum Medicaid beyond 60 days) as of January 2020. See **eTable1** for the FFCRA-associated postpartum Medicaid eligibility change for each sampled state.

To measure the association between state FFCRA-associated postpartum Medicaid eligibility change and the outcomes, we estimated survey-weighted linear regression models for each outcome (Y_{ist}) where *i* indexes individual, *s* state, and *t* month:

$$Y_{ist} = \delta_s + \delta_t + \beta_1(FFCRA\_Eligibility\_Change_s * Post\_FFCRA_t) + \beta_x X_{st} + \beta_x X_{ist} + \varepsilon_{ist}$$
(Equation 1)

The primary coefficient of interest (β_1) is the interaction between an indicator for whether a live birth occurred in the post-FFCRA policy period (January 2020-Dec 2021; $Post\_FFCRA_t$) and a 10 percentage point change in the Medicaid eligibility threshold in each state ($FFCRA\_Eligibility\_Change_s$). The coefficient of interest (β_1) represents the association between each outcome and a 10 percentage-point (p.p.) increase in the postpartum Medicaid eligibility threshold. We also provide the estimated percent change in the outcome by dividing the percentage-point change by the baseline prevalence of the outcome. By providing both the percentage-point change and percent change, we provide an estimate of both the absolute and relative impact of the policy.

All regression models include state fixed effects (δ_s) to control for time-invariant differences in the outcomes across states and year fixed effects (δ_t) to control for time effects common across all states. In adjusted analyses, we also control for a vector of individual-level variables (X_{ist}) including maternal age, race-ethnicity, marital status, education, primary survey language, household income, geography (rural/urban resident), parity, chronic conditions, and month of survey completion. The PRAMS household income variable is based on categorical income-bands ($0-$16,000 and $4,000 bins up to $60,000). To conservatively estimate poverty status, we assumed the maximum value of the specified income band and then converted this income to a percentage of the FPL based on year, state, and household size. Adjusted models also include state-month level control variables (X_{st}) economic conditions (unemployment rate), COVID-19 impact (case rate, death rate) and COVID-19 policies (stay at home orders, ACA special enrollment periods, suspensions of elective medical procedures). **eTable 7** provides definitions and data sources for state-month level variables. We calculated heteroskedasticity-robust standard errors clustered at the state level to account for within-state correlation of the error terms and the state-level nature of the intervention.

b) Race-Ethnicity Stratified Subgroup Analysis

We explored differential associations by maternal race and ethnicity using stratified DID models (Equation 1) by race-ethnicity (to return estimates and statistical tests of the effect of the policy within groups). Due to small sample sizes, we only considered three race-ethnicity subgroups: white non-Hispanic, Black non-Hispanic and Hispanic. Also as a result of small sample sizes

and limited statistical power, we did not formally test whether the associations were statistically different between race-ethnicity subgroups.

c) Placebo Event Studies

Causal interpretations of the estimates from Equation 1 rest on "parallel trend"-type assumptions: that the changes in the outcomes from pre- to post-FFCRA policy adoption would have been the same across states if not for state-level differences in the pre-post FFCRA eligibility change, conditional on adjusting for state and year fixed effects and covariates. While we cannot directly test these assumptions, as it would require observing the counterfactual where the policies were not enacted, evidence of parallel trends in pre-policy data is supportive of the internal validity of our study designs. Importantly, our study design does not require that states with differing eligibility changes have similar outcome "levels" (i.e., outcome means) prior to the policy. Rather, it only requires that the outcomes would have continued along a similar trend (if not for the policy) across states with differing eligibility changes.

To assess these assumptions, first, we examined the sample characteristics by tertile of the FFCRA-associated postpartum Medicaid eligibility change (**eTable 2**). Similar characteristics across tertiles are supportive of the theory that the outcomes would have evolved in the same way for individuals in states with different levels of the FFCRA-associated postpartum Medicaid eligibility change, if not for the FFCRA implementation. In general, we see that the sample characteristics are reasonably similar across tertiles. One notable exception is that individuals in states in the highest tertile of eligibility change were more likely to speak English and live in a rural area.

Second, since linear trend tests in the pre-policy period can be underpowered and impose a restriction on the form of the violation of the parallel trends assumption (i.e. assuming a violation must be linear), we conducted event studies, which offer a more flexible test of differential pre-policy trends which does not assume linearity. In this test, for each outcome we estimate a survey-weighted regression that includes a set of interactions between the FFCRA-associated Medicaid eligibility change and each year expansion (excluding 2019, the last year prior to the FFCRA as the reference year):

$$Y_{ist} = \delta_s + \delta_t + \sum_{j \neq k} \delta_j (Expansion_s \cdot I(t = j)) + \beta_x X_i + \Omega\, Year_i + \Omega\, Month_i + \varepsilon_{ist}$$

(Equation 3)

where k indicates the last pre-policy year (2019) and all other variables are defined as in Equation 1. The coefficients, δ_j, where $j < k$ are essentially placebo tests for whether the FFCRA-associated Medicaid eligibility change was associated with changes in the outcomes in the period prior to the policy (relative to the last pre-policy year). We also jointly tested the null hypothesis that both of the two pre-policy interaction terms (δ_j where $j < k$) are equal to zero using an F-test. Failure to reject the null hypothesis that these pre-period interaction coefficients are not significantly different than zero is supportive of the parallel trends assumption. **eFigure 2** plots the interaction term coefficients and 95% confidence intervals for all study years (relative to the reference year, 2019). **eTable 3** shows the F-statistics and p-values for the pre-period event study coefficients. The event study plots and tests show significant differential trends in commercial coverage in the pre-policy period. The event study also reveals significant pre-policy trends in effective birth control and still breastfeeding.

Overall, our assessment suggests that the validity of the study design differs across outcomes. For uninsurance, postpartum visits, LARC, and depressive symptoms, differential pre-policy differences across states with varying levels of Medicaid eligibility changes were small and not

significant, suggesting that the assumptions are likely to be valid for these outcomes. However, the event study plots showed a positive trend in Medicaid coverage in the pre-policy period. While the joint test of the event study coefficients were not statistically significant for the Medicaid outcome, we cannot rule out potential for some bias. For commercial coverage, we find consistent evidence of a negative pre-policy trend associated with greater levels of FFCRA-eligibility change. Thus, our estimates of changes for commercial coverage should be interpreted with caution. Similar caution may be warranted for still breastfeeding and effective birth control, though the magnitude of the event study coefficients were small. We have included notes in all regression tables to flag these potential violations of the assumptions of the design.

© 2024 Daw JR et al. *JAMA Health Forum.*

eTable 1. *FFCRA*-associated postpartum Medicaid income eligibility changes by state

Study Sample, N=21 states			
State	Pregnant Women	Low-Income Parents/Adults	Eligibility Change
AL	146	18	128
CO	265	138	127
CT	263	160	103
DE	217	138	79
GA	225	35	190
IL	213	138	75
KS	171	38	133
LA	214	138	76
MA	205	138	67
MI	200	138	62
MT	162	138	24
NJ	205	138	67
NM	255	138	117
NY	223	138	85
ND	162	138	24
PA	220	138	82
SD	138	48	90
VT	213	138	75
WA	198	138	60
WI	306	100	206
WY	159	53	106
**Mean**	208	114	**94**
**Median**	213	138	**82**

Not In Study Sample, N=29 states + DC			
State	Pregnant Women	Low-Income Parents/Adults	Eligibility Change
AK	205	138	67
AZ	161	138	23
AR	214	138	76
CA	322	138	184
DC	324	221	103
FL	196	31	165
HI	196	138	58
ID	138	138	0
IN	218	138	80
IA	380	138	242
KY	200	138	62
ME	214	138	76
MD	264	138	126
MN	283	138	145
MS	199	26	173
MO	305	21	284
NE	202	63	139
NV	165	138	27
NH	201	138	63
NC	201	41	160
OH	205	138	67
OK	210	41	169
OR	190	138	52
RI	258	138	120
SC	199	67	132
TN	255	94	161
TX	207	17	190
UT	144	138	6
VA	205	138	67
WV	305	138	167
**Mean**	226	113	**113**
**Median**	205	138	**112**

All States + DC			
**Mean:**	218	113	**105**
**Median:**	205	138	**85**

Source: Kaiser Family Foundation, 2020 Medicaid income eligibility limits.

© 2024 Daw JR et al. *JAMA Health Forum.*

eTable 2. Sample characteristics by tertile of state FFCRA-associated postpartum Medicaid eligibility change for postpartum people with Medicaid-paid births

**Characteristic**	**Tertile 1, N=18067**		**Tertile 2, N=15243**		**Tertile 3, N=14406**	
Age						
<20	1240	6.4%	1018	7.0%	1261	9.0%
20-24	4327	25.3%	3692	25.6%	3990	28.6%
25-29	5603	30.8%	4468	29.9%	4403	31.2%
30-34	4264	23.1%	3620	22.2%	3039	20.4%
≥35	2633	14.3%	2445	15.3%	1713	10.8%
Race-Ethnicity						
White non-Hispanic	4308	38.5%	3318	32.2%	4510	37.7%
Black non-Hispanic	5055	21.3%	4810	28.0%	3649	30.5%
Hispanic	2835	21.4%	2451	14.6%	3633	20.2%
Other	4392	16.9%	4581	24.2%	2558	11.0%
Missing	1477	1.8%	83	1.0%	56	0.6%
Education						
<High School	3502	19.9%	3444	21.1%	2969	20.5%
High School	6571	38.6%	5648	39.0%	5534	42.4%
>High School	7757	40.0%	6040	39.3%	5808	36.5%
Missing	237	1.5%	111	0.6%	95	0.7%
Household Income (% FPL)						
<100%	7444	37.8%	6435	39.1%	6442	40.9%
100-149%	3299	19.1%	2412	15.7%	2754	19.5%
150-204%	2145	12.5%	1559	10.3%	1610	11.9%
≥205%	2399	13.9%	1721	12.1%	1838	13.6%
Missing	2780	16.8%	3116	22.9%	1762	14.2%
Married	6220	36.8%	4946	33.7%	4970	35.6%
Rural Resident	4103	13.1%	1978	9.0%	4297	24.1%
Survey Language						
English	16282	86.8%	12817	83.1%	13242	90.9%
Spanish or Chinese	1785	13.2%	2426	16.9%	1164	9.1%
Primary Payer for Childbirth						
Medicaid	18067	100.0%	15243	100.0%	14406	100.0%
Commercial	-	-	-	-	-	-
Uninsured	-	-	-	-	-	-
Primiparous	5882	32.0%	5047	34.6%	4642	33.1%
Any Chronic Conditions*	4658	23.2%	3451	20.7%	4000	23.6%
Missing	105	0.6%	132	1.2%	110	0.7%
**Mean FPL Change**	65%		83%		161%	

Notes: Estimates shown are unweighted frequencies and survey-weighted percentages. Missing not shown for variables with <1% missing values for all tertiles. *Self-reported hypertension, diabetes, or depression in the 3 months prior to pregnancy.

© 2024 Daw JR et al. *JAMA Health Forum.*

eFigure 1. Unadjusted scatterplots and linear relationship between FFCRA postpartum Medicaid eligibility change and pre-post FFCRA postpartum outcomes by state among postpartum people with Medicaid-paid births

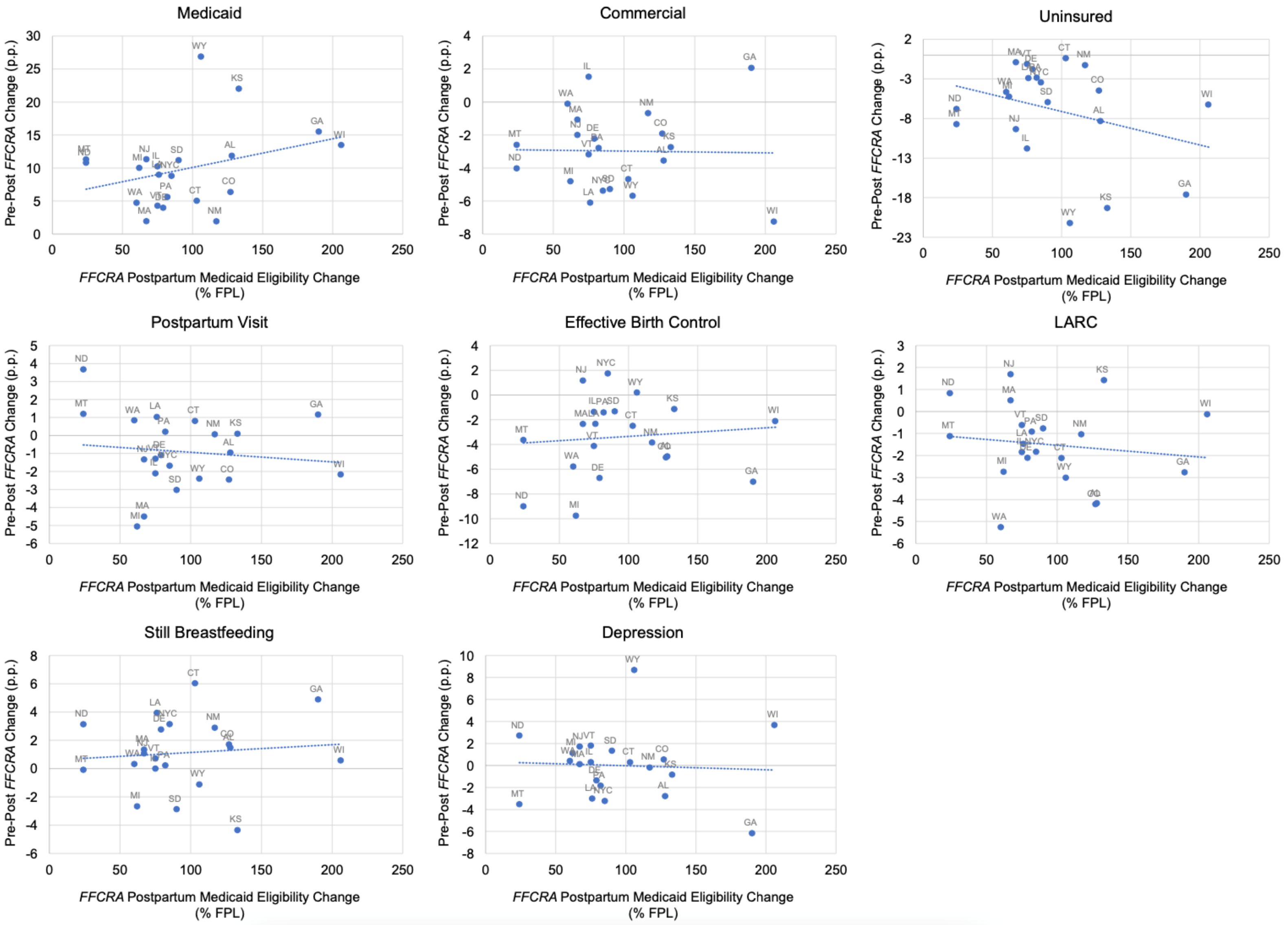

© 2024 Daw JR et al. *JAMA Health Forum.*

eFigure 2. Event study plots

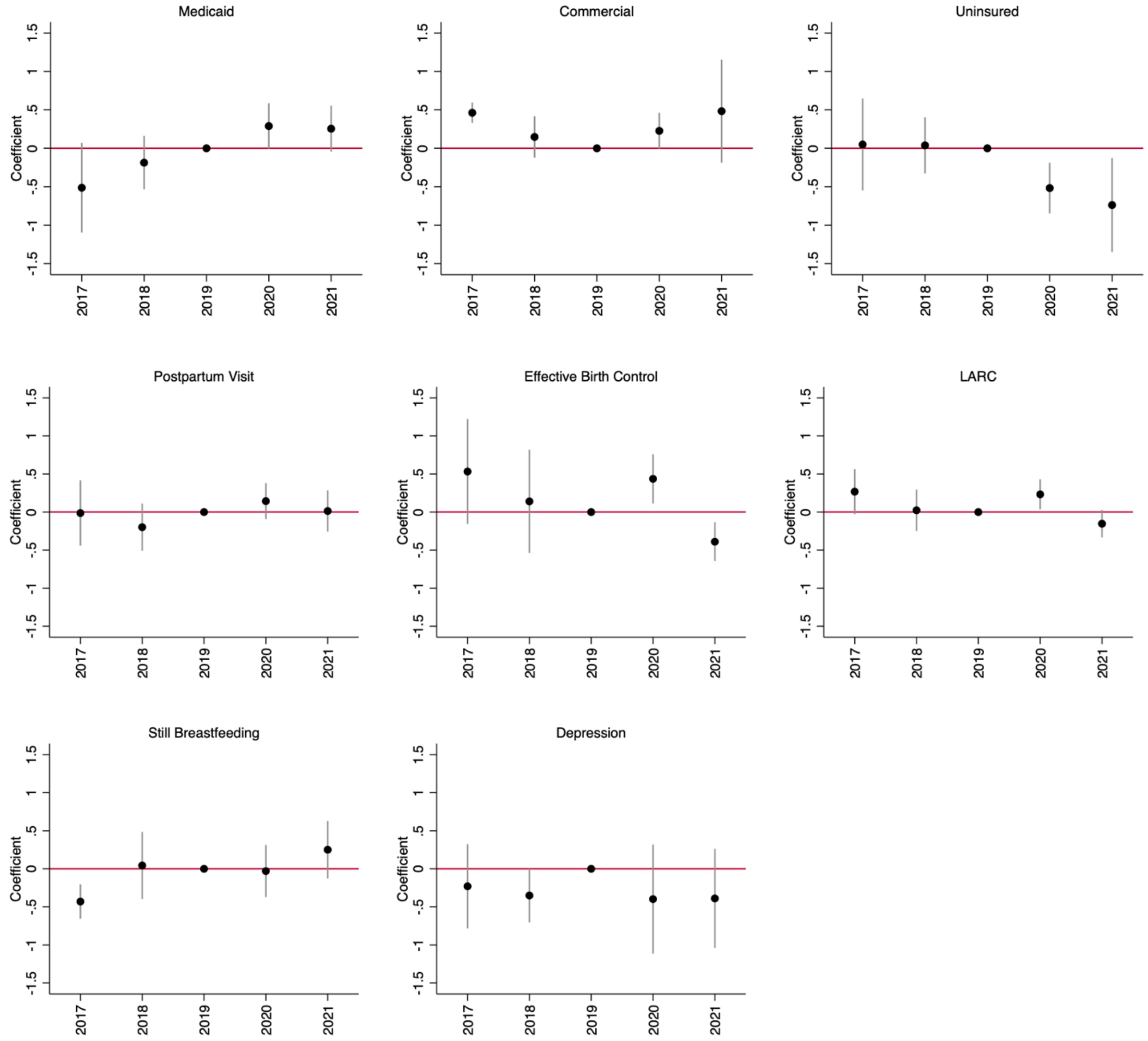

© 2024 Daw JR et al. *JAMA Health Forum.*

eTable 3. F-tests for pre-period event study coefficients

Postpartum Outcome	Joint Test of Event Study Coefficients	
	**F-Statistic**	**P-Value**
Insurance Status		
Medicaid	1.69	0.210
Commercial	27.37	<0.0001*
Uninsured	0.03	0.971
Postpartum Visit	0.99	0.388
Effective Birth Control	5.82	0.0102*
LARC	2.17	0.141
Still Breastfeeding	8.24	0.0025*
Depressive Symptoms	2.41	0.116

Notes: F-statistics and p-values from the test of the null hypothesis that all of the pre-period coefficients (which measure the association between an interaction of each pre-policy year and the *FFCRA*-associated postpartum Medicaid eligibility change and the outcomes) are equal to zero. *Statistically significant at 0.05 significance level.

© 2024 Daw JR et al. *JAMA Health Forum.*

eTable 4. Difference-in-differences estimates stratified by race-ethnicity

	White NH N=12,136		Black NH N=13,514		Hispanic N=8,919	
**Postpartum Outcomes**	Baseline	Adjusted DID	Baseline	Adjusted DID	Baseline	Adjusted DID
Insurance Status						
Medicaid	66.3	8.3 (2.9, 13.6)*	69.2	4.2 (-1.5, 9.9)[c]	55.2	2.7 (-4.5, 9.9)
Commercial	20.8	4.2 (-0.6, 9.0)[c]	20.7	2.8 (-2.2, 7.8)[c]	17.0	-2.2 (-7.5, 3.2)
Uninsured	12.9	-12.5 (-16.6, -8.4)*[c]	10.1	-7.0 (-11.1, -3.0)*[c]	27.8	-0.5 (-7.7, 6.6)
Postpartum Visit	87.4	-0.2 (-4.3, 3.9)	85.0	1.0 (-3.2, 5.2)	85.5	2.1 (-3.9, 8.0)
Effective Contraception	57.4	-0.4 (-6.5, 5.7)[c]	57.5	-5.8 (-12.1, 0.6)[c]	60.9	-1.6 (-10.1, 6.8)
LARC	17.5	2.7 (-2.3, 7.6)	16.8	-3.0 (-7.9, 1.8)	26.8	-2.2 (-9.1, 4.7)
Still Breastfeeding	41.2	1.2 (-4.5, 6.9)[c]	27.0	1.2 (-4.1, 6.6)	42.4	12.8 (4.7, 20.8)*
Depressive Symptoms	17.8	-1.6 (-6.1, 3.0)[c]	20.3	-2.5 (-6.9, 1.9)[c]	12.4	-0.6 (-5.9, 4.7)

Notes: Adjusted models include age, education, urban/rural residence, marital status, survey language, parity, chronic conditions, postpartum month of survey, household income, state-month level unemployment and state-month level COVID-19 covariates (COVID case rate, COVID death rate, any state at home order, ACA special enrollment period, suspension of elective medical procedures). *Statistically significant at 0.05 significance level.
[c] Event study analyses and corresponding F test suggests the parallel-trend assumption may be violated. These results should be interpreted with caution.

© 2024 Daw JR et al. *JAMA Health Forum.*

eFigure 3 Percent change in postpartum insurance status associated with a 100% FPL increase in postpartum Medicaid eligibility by race-ethnicity

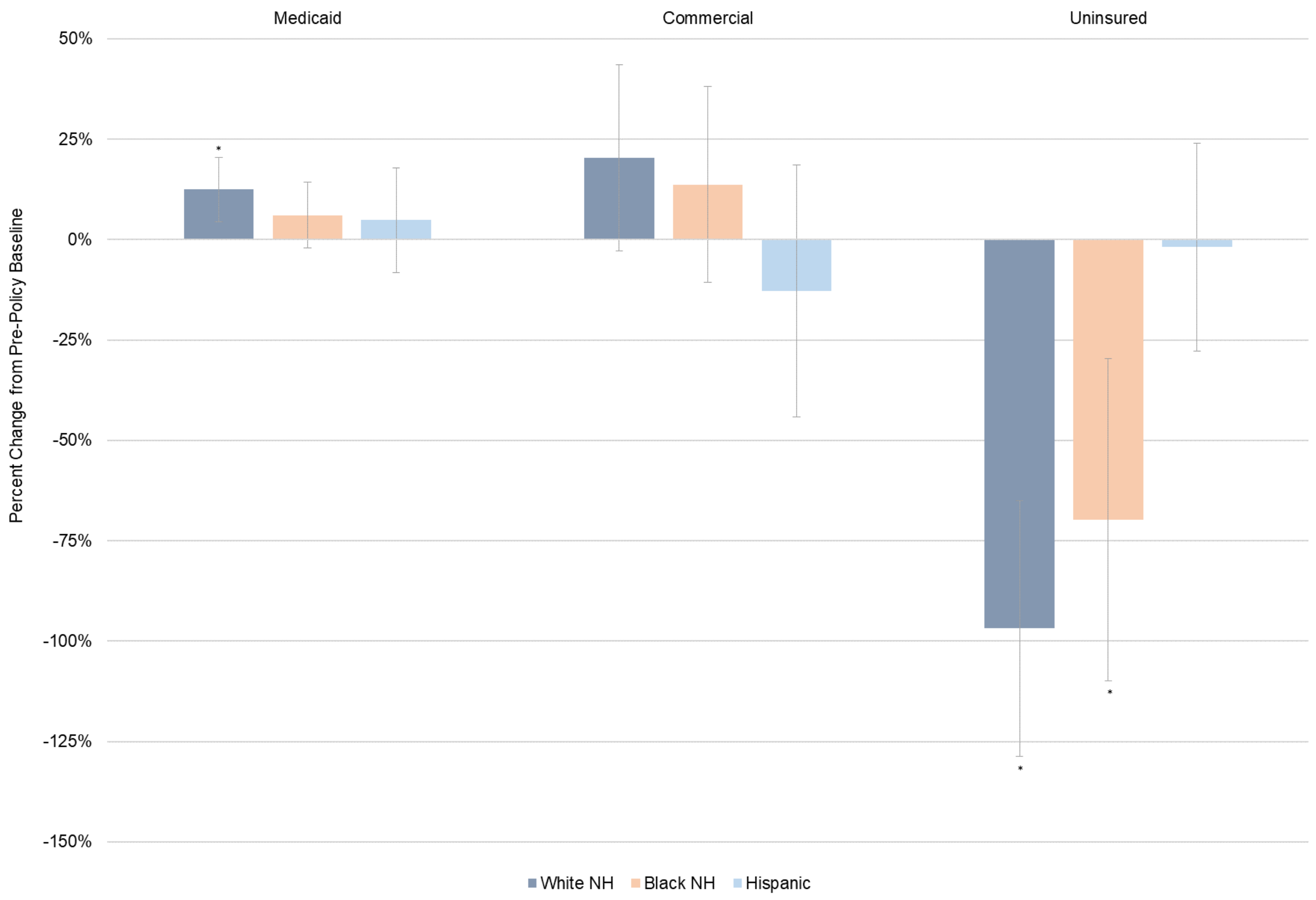

Notes: Percent change is calculated as the adjusted difference-in-difference estimate associated with a 100% FPL change in postpartum Medicaid eligibility relative to the pre-policy baseline (2017-2019). Postpartum insurance is measured at the time of the PRAMS survey (mean: 4 months after a live birth). *Statistically significant ($p<0.05$).

© 2024 Daw JR et al. *JAMA Health Forum.*

eTable 5. Difference-in-differences estimates stratified by pre-FFCRA income eligibility generosity for pregnant individuals

	Less generous pre-FFCRA pregnancy eligibility (n=20,589)			More generous pre-FFCRA pregnancy eligibility (n=27,127)		
**Postpartum Outcomes**	Baseline	Unadjusted DID	Adjusted DID	Baseline	Unadjusted DID	Adjusted DID
Insurance Status						
Medicaid	63.6	9.6 (-0.9, 20.0)	9.3 (1.5, 17.0)*[c]	62.9	5.5 (1.9, 9.1)*	5.0 (0.6, 9.4)*
Commercial	19.5	-1.2 (-4.6, 2.1)	-0.4 (-2.5, 1.7)	20.6	1.7 (-4.9, 8.3)	1.7 (-3.9, 7.3)[c]
Uninsured	16.9	-8.3 (-18.7, 2.0)	-8.9 (-18.1, 0.4)[c]	16.5	-7.2 (-16.8, 2.4)	-6.7 (-16.1, 2.7)
Postpartum Visit	85.0	1.2 (-3.7, 6.1)	1.0 (-3.9, 5.9)[c]	86.2	0.8 (-1.7, 3.3)	0.3 (-2.0, 2.5)
Effective Contraception	57.8	2.8 (-4.5, 10.1)	1.8 (-5.2, 8.7)[c]	56.2	-4.1 (-7.7, -0.5)*	-5.4 (-10.1, -0.6)*[c]
LARC	18.7	-0.6 (-7.3, 6.1)	-1.7 (-6.8, 3.4)	19.9	-0.5 (-2.0, 1.1)	-0.8 (-3.1, 1.4)[c]
Still Breastfeeding	40.1	-0.1 (-6.3, 6.0)	3.4 (-0.9, 7.7)	37.1	1.5 (-1.7, 4.7)	2.1 (-1.6, 5.8)
Depressive Symptoms	18.5	-3.2 (-7.1, 0.8)	-2.2 (-5.3, 0.9)	16.4	-1.1 (-7.3, 5.1)	-1.2 (-6.3, 3.9)

Notes: [a] Less generous pregnancy eligibility was defined as an income eligibility threshold below the median of the 21 states included in our primary analysis (i.e., 213% FPL). This includes: AL, KS, MA, MI, MT, ND, NJ, SD, WA, and WY, which had pregnancy eligibility thresholds ranging from 138% to 205% of the FPL.
[b] More generous pregnancy eligibility was defined as an income eligibility threshold at or above the median of the 21 states included in our primary analysis (i.e., 213% FPL). This includes: CO, CT, DE, GA, IL, LA, NM, NY, PA, VT, and WI, which had pregnancy eligibility thresholds ranging from 213% to 306% of the FPL.
[c] Event study analyses and corresponding F test suggests the parallel-trend assumption may be violated. These results should be interpreted with caution.

© 2024 Daw JR et al. *JAMA Health Forum.*

eTable 6. Difference-in-differences estimates for additional contraceptive categories

Postpartum Contraceptive Method	Pre-Policy Baseline	Percentage Point Change Associated with a 100% FPL Increase in Postpartum Medicaid Eligibility	
		Unadjusted	*Adjusted*
Effective contraception	56.8	-1.7 (-5.7, 2.3)	-3.1 (-7.4, 1.3)[c]
Tubal ligation	10.2	0.3 (-0.7, 1.3)	-0.3 (-1.4, 0.8)
LARC	19.5	-0.4 (-2.2, 1.3)	-0.9 (-2.8, 1.0)
Short-acting hormonal methods	27.2	-2.1 (-5.1, 0.9)[c]	-2.5 (-5.7, 0.8)[c]
Condoms only	9.5	1.1 (-2.8, 5.0)	1.3 (-2.7, 5.3)
No method or only non-hormonal, non-barrier	29.7	-0.1 (-2.4, 2.2)	0.8 (-1.4, 3.0)

Notes: Long acting reversible contraceptives (LARC) includes intrauterine devices (IUDs) and contraceptive implants. Short-acting hormonal methods include the contraceptive pill, patch, ring, and injection. Non-hormonal, non-barrier methods include abstinence, natural family planning (or rhythm method), and withdrawal.
[c] Event study analyses and corresponding F test suggests the parallel-trend assumption may be violated. These results should be interpreted with caution.

© 2024 Daw JR et al. *JAMA Health Forum.*

eTable 7. Definitions and data sources for state-month level COVID covariates

Covariate	Definition	Data Sources
COVID-19 case rate	The number of COVID-19 cases per 100,000 population per month for each state.	*Numerator:* The New York Times[1]<br>*Denominator:* American Community Survey[2]
COVID-19 death rate	The number of COVID-19 deaths per 100,000 population per month for each state.	*Numerator:* The New York Times[1]<br>*Denominator:* American Community Survey[2]
Stay home order	Indicates months in which a stay home order was in effect for each state. This includes stay home orders with and without explicit restriction of movement for the public.	COVID-19 US state policy database[3]
ACA special enrollment period	Indicates months in which the ACA health insurance exchanges (i.e., the Marketplaces) were reopened through a special enrollment period (SEP) for each state.	*2020 SEP start dates:*<br>COVID-19 US state policy database[3]<br><br>*2020 SEP end dates:*<br>Local or state news releases[4-15]<br><br>*2021 SEP start and end dates:*<br>US Department of Health and Human Services[16]
Suspension of elective medical procedures	Indicates months in which elective medical procedures were suspended in each state.	COVID-19 US state policy database[3]
Unemployment rate	The seasonally adjusted percent of the labor force that was unemployed for each month in each state.	US Bureau of Labor Statistics[17]

© 2024 Daw JR et al. *JAMA Health Forum.*

eReferences

1. The New York Times. Coronavirus (COVID-19) data in the United States. Accessed July 14, 2023. Available at: https://github.com/nytimes/covid-19-data
2. U.S. Census Bureau. Annual estimates of the resident population for the US, regions, states, District of Columbia, and Puerto Rico: April 1, 2020 to July 1, 2022. Accessed July 14, 2023. Available at: https://www.census.gov/data/tables/time-series/demo/popest/2020s-state-total.html
3. Raifman J, Nocka K, Jones D, Bor J, Lipson S, Jay J, and Chan P. COVID-19 US state policy database. Accessed July 14, 2023. Available at: https://github.com/USCOVIDpolicy/COVID-19-US-State-Policy-Database
4. Department of Managed Health Care. APL 20-029 - Extension of special enrollment period to August 31, 2020. Published July 31, 2020. Available at: https://www.dmhc.ca.gov/Portals/0/Docs/OPL/APL 20-029 - Extension of Special Enrollment Period to August 31, 2020 (7_31_20)_1.pdf
5. Colorado Department of Regulatory Agencies. COVID-19 special enrollment period for health insurance extended to April 30. Published April 2, 2020. Available at: https://doi.colorado.gov/press-release/covid-19-special-enrollment-period-for-health-insurance-extended-to-april-30
6. Access health CT. Access Health CT extends new special enrollment period for the uninsured. Published April 2, 2020. Available at: https://agency.accesshealthct.com/access-health-ct-extends-new-special-enrollment-period-for-the-uninsured
7. Health Benefit Exchange Authority. DC Health Link expands opportunities to get covered during public health emergency. Published April 6, 2020. Available at: https://hbx.dc.gov/release/dc-health-link-expands-opportunities-get-covered-during-public-health-emergency
8. Maryland Health Benefit Exchange. Coronavirus emergency special enrollment period deadline extended to July 15. Published June 18, 2020. Available at: https://insurance.maryland.gov/COVID-19/Documents/Coronavirus-SEP-July-15-Extension-Release-6182020.pdf
9. Massachusetts Health Connector. COVID-19 special enrollment period: final enrollment results. Published 2020. Available at: https://www.mahealthconnector.org/wp-content/uploads/Health-Connector-COVID-19-SEP-Brief.pdf
10. Office of the Governor. Governor Tim Walz, MNsure announce special enrollment period to increase health care access during COVID-19. Published March 20, 2020. Available at: https://mn.gov/governor/newsroom/press-releases/#/detail/appId/1/id/424211
11. Office of the Governor. ICYMI: Silver State Health Insurance Exchange announces special enrollment period on Nevada Health Link. Published March 19, 2020. Available at: https://gov.nv.gov/layouts/full_page.aspx?id=302028

© 2024 Daw JR et al. *JAMA Health Forum.*

12. Office of the Governor. Governor Cuomo announces special enrollment for uninsured extended through the end of 2020. Published September 16, 2020. Available at: https://www.governor.ny.gov/news/governor-cuomo-announces-special-enrollment-uninsured-extended-through-end-2020-0

13. HealthSource RI. Health coverage special enrollment period (SEP) extended through April 30, 2020. Published April 13, 2020. Available at: https://healthsourceri.com/sep-extended/

14. Department of Vermont Health Access. Special enrollment period for uninsured Vermonters extended to May 15th. Published April 14, 2020. Available at: https://legislature.vermont.gov/Documents/2020/WorkGroups/House Health Care/COVID-19/W~Adaline Strumolo~Special Enrollment Period for Uninsured Vermonters Extended to May 15th~4-22-2020.pdf

15. Washington State Commission on Hispanic Affairs. Washington Healthplanfinder extends current special enrollment period, gives extra month for uninsured to secure health coverage. Published April 1, 2020. Available at: https://www.cha.wa.gov/news/2020/4/1/washington-healthplanfinder-extends-current-special-enrollment-period-gives-extra-month-for-uninsured-to-secure-health-coverage

16. US Department of Health and Human Services. 2021 Final Marketplace Special Enrollment Period Report. Accessed on July 17, 2023. Available at: https://www.hhs.gov/sites/default/files/2021-sep-final-enrollment-report.pdf

17. US Bureau of Labor Statistics. State unemployment rates over the last 10 years, seasonally adjusted. Accessed on July 12, 2023. Available at: https://www.bls.gov/charts/state-employment-and-unemployment/state-unemployment-rates-animated.htm

© 2024 Daw JR et al. *JAMA Health Forum.*
