# Supplementary material for: Continuous Medicaid Eligibility During the COVID-19 Pandemic and Postpartum Coverage, Health Care, and Outcomes
Source: JAMA Health Forum. 2024 Mar 8;5(3):e240004. doi: 10.1001/jamahealthforum.2024.0004 (PMC10924249; doi:10.1001/jamahealthforum.2024.0004)
Supplement: Supplement 2. — Data Sharing Statement [file jamahealthforum-e240004-s002.pdf]

Data Sharing Statement

Data
Data available: No

Additional Information
Explanation for why data not available: Access to the PRAMS is governed by the U.S. Centers for Disease Control and Prevention. Researchers may gain access to it by applying at https://www.cdc.gov/prams/prams-data/researchers.htm
